# Direct cadaverine production from cellobiose using β-glucosidase displaying *Escherichia coli*

**DOI:** 10.1186/2191-0855-3-67

**Published:** 2013-11-08

**Authors:** Naoki Ikeda, Mari Miyamoto, Noriko Adachi, Mariko Nakano, Tsutomu Tanaka, Akihiko Kondo

**Affiliations:** 1 Department of Chemical Science and Engineering, Graduate School of Engineering, Kobe University, 1-1 Rokkodai-cho, Nada, Kobe 657-8501, Japan

**Keywords:** Cadaverine, *E. coli*, Cell surface display, Cellobiose

## Abstract

In this study, we demonstrate the one-step production of cadaverine (1,5-diaminopentane) from cellobiose using an *Escherichia coli* strain displaying β-glucosidase (BGL) on its cell surface. L-lysine decarboxylase (CadA) derived from *E. coli* and BGL from *Thermobifida fusca* YX (Tfu0937) fused to the anchor protein Blc from *E. coli* were co-expressed using *E. coli* as a host. The expression of CadA was confirmed by Western blotting and BGL activity on the cell surface was evaluated using pNPG as a substrate. Growth on cellobiose as the sole carbon source was also achieved. The OD600 value of the BGL and CadA co-expressing strain was 8.0 after 48 h cultivation, which is higher than that obtained by growth on glucose (5.4 after 48 h cultivation). The engineered strain produced cadaverine from cellobiose more effectively than from glucose: 6.1 mM after 48 h from 28 g/L of consumed cellobiose, vs. 3.3 mM from 20 g/L of consumed glucose.

## Introduction

Cadaverine, also known as 1,5-diaminopentane, has many industrial applications. Similar to putrescine (1,4-diaminobutane), cadaverine serves as a component of polymers such as polyamides and polyurethane, and as a component of chelating agents and other additives. Cadaverine is particularly relevant in the production of bio-polyamides derived from renewable feedstocks as a replacement for conventional polyamides derived from petrochemicals. The petrochemical route suffers from the limited supply and rising price of fossil fuels, as well as from low eco-efficiency. Polyamide 54 is made by the polycondensation of cadaverine and succinic acid. Polyamide 54 holds promise as a bio-based alternative to conventional petroleum-based polyamides that are currently produced on the order of 3.5 million tons annually (Kind et al. 2010; Mimitsuka et al. 2007).

In the Gram-negative bacterium *Escherichia coli*, cadaverine is biosynthesized directly from L-lysine by two kinds of L-lysine decarboxylases: a constitutive one encoded by the *ldcC* gene, and an inducible one produced at low pH encoded by the *cadA* gene. The level of cadaverine in *E. coli* is regulated by biosynthesis, degradation, uptake, and export (Soksawatmaekhin et al. 2004). It was recently shown that *E. coli* can tolerate a higher concentration of cadaverine, just slightly lower than that tolerated by a wild-type *Corynebacterium glutamicum* strain (Mimitsuka et al. 2007; Qian et al. 2011). This high tolerance suggests that microorganisms can be metabolically engineered to overproduce cadaverine to industrially useful levels.

*E. coli* is an attractive bacterium due to its fast growth in inexpensive media, demonstrated scale-up processes, well-understood genetic background and metabolism, and the availability of established metabolic engineering tools. In particular, this bacterium has recently been metabolically engineered to produce biomass-derived biofuels and chemicals (Atsumi et al. 2008; Lee et al. 2007; Park et al. 2008; Park et al. 2007; Qian et al. 2009; Steen et al. 2010; Tyo et al. 2009). A cadaverine overproducing *E. coli* strain has been engineered by the introduction of the cadaverine biosynthesis, degradation, and utilization pathways (Na et al. 2013; Qian et al. 2011). This metabolically engineered *E. coli* strain could constitutively produce cadaverine to a high concentration in glucose mineral salts medium. However, there are no reports of the use of *E. coli* strain to produce cadaverine using cellobiose as a carbon source.

Lignocellulosic biomass is one of the most attractive bioresources because of its abundance and the fact that it is renewable. The use of lignocellulosic biomass has therefore recently garnered attention (Sánchez and Cardona 2008). However, in order to use lignocellulose as a bioresource, costly and complex intermediate chemical steps such as milling and hydrolysis treatment, followed by fermentation, are required (Sánchez and Cardona 2008). It is therefore important to develop an efficient and cost-effective method to convert lignocellulosic biomass into simple fermentable sugars.

The efficient degradation of cellulose requires a synergistic reaction of the cellulolytic enzymes endoglucanase (EG), cellobiohydrolase (CBH), and β-glucosidase (BGL). The cellulose is degraded by EG and CBH, resulting in cellobiose and some cellooligosaccharides, which can be converted to glucose by BGL. BGL catalyzes the final step in cellulose degradation, as well as stimulating cellulose hydrolysis by relieving the cellobiose-mediated inhibition of EG and CBH. To date, there have been only a few reports on the production of valuable chemicals such as isopropanol (Soma et al. 2012) from cellobiose or cello-oligosaccharides by directly using *E. coli*, and no reports of the production of cadaverine from cellobiose using *E. coli*. The use of BGL-expressing *E. coli* may lead to reduced usage of cellulases, thereby reducing the significant costs of hydrolysis in the production of cellulosic biomass-derived products.

Here, we demonstrate direct cadaverine production using cellobiose as a carbon source. We employed cell surface display, which is a powerful tool for improving the activity of a displayed protein (Lee et al. 2003; Tanaka et al. 2011). We previously reported a cell surface display system in *E. coli* using Blc as an anchor (Tanaka et al. 2011) and the direct assimilation of cellobiose using defined minimum medium. In the present study, using *E. coli* as a host, we demonstrate the direct production of cadaverine from cellobiose.

## Materials and methods

### Bacterial strains and media

Table 1 shows the strains and plasmids used in this study. *E. coli* JCM20137 was used as the host strain. This bacterium, and its derivative possessing a plasmid, were grown in Luria-Bertani medium (10 g/l tryptone, 5 g/l yeast extract, 5 g/l sodium chloride) containing 100 μg/ml ampicillin at 37°C. For cadaverine fermentation, R/2 (Qian et al. 2011) medium containing 100 μg/ml ampicillin and 40 g/l glucose or 40 g/l cellobiose was used. R/2 medium (pH 6.80) contains 3.17 g/l of NaNH_4_HPO_4_ 4H_2_O, 6.75 g/l of KH_2_PO_4_, 0.85 g/l of citric acid, 0.7 g/l of MgSO_4_ 7H_2_O and 5 ml/l of a trace metal stock solution (Lee and Chang 1993).

**Table 1:** Strains, plasmids, and primers used in this study

**Strain, plasmid, or primer**	**Relevant phenotype, description, or sequence (5′-3′)**	**Source or reference**
Strains		
*Escherichia coli*		
NovaBlue	endA1 hsdR17 (rK12^-^ mK12^+^) supE44 thi-1 recA1 gyrA96 relA1 lac[F’ proAB^+^ lacI^q^ZΔM15::Tn10 (Tet^r^)]; host for DNA manipulation	Novagen
JCM20137		National Institute of Genetics, Japan
		Japan Collection of Microorganisms, RIKEN BRC
Jm-cadA	JCM20137 strain harboring pHLA-cadA vector	This study
Jm-blc-Tfu	JCM20137 strain harboring pHLA-blc-Tfu vector	This study
Jm-cadA-blc-Tfu	JCM20137 strain harboring pHLA-cadA-blc-Tfu vector	This study
Genomic DNA		
*Thermobifida fusca* YX	ATCC BAA-629D-5	ATCC
Plasmids		
pHLA	Cell surface display vector containing pgsA gene under HCE promoter control, ampicillin resistance marker	Tanaka et al. 2011
pHLA-cadA	Vector for CadA expression	This study
pHLA-blc-Tfu	Vector for BGL (Tfu0937 from *T. fusca*) expression using blc anchor protein	Tanaka et al. 2011
pHLA-cadA-blc-Tfu	Vector for CadA and BGL (Tfu0937 from *T. fusca*) coexpression using blc anchor protein	This study
Oligonucleotide primers		
CadA_F	agcagatctgatgaacgttattgcaatattgaatcacatggggg	
CadA-FLAG_R	ccagtcgacttatttatcgtcatcatctttataatcttttttgctttcttc	
HCE-blc-Tfu-term_F	cgccgtagcgccgatggtagtgtggggtctccccatgcgag	
HCE-blc-Tfu-term_R	ggagagatcgcggccgccatggggtcaggtgggaccaccgcgctac	
pHLA-cadA_F	gcggccgcgatctctccttcacagattcccaatctcttgtt	
pHLA-cadA_R	ctcgcatggggagaccccacactaccatcggcgctacggcg	

### Plasmid construction

Oligonucleotide primers used for plasmid construction are listed in Table 1. Polymerase chain reaction (PCR) was conducted using KOD FX DNA polymerase (TOYOBO Co., Ltd., Osaka, Japan).

The plasmid for lysine decarboxylase (CadA) expression was constructed as follows. The gene encoding CadA was amplified by PCR using CadA_F and CadA-FLAG_R primers with *E. coli* Novablue genomic DNA as the template. The amplified fragment was introduced into the *Bgl*II and *Sal*I sites of plasmid pHLA (Narita et al. 2006). The resulting plasmid was designated pHLA-cadA. The plasmid for both CadA and BGL expression was constructed as follows. The gene encoding the HCE promoter Blc-Tfu0937 terminator region was amplified by PCR using HCE-blc-Tfu-term_F and HCE-blc-Tfu-term_R primers with plasmid pHLA-blc-Tfu (Tanaka et al. 2011) as a template. The amplified fragment was ligated into plasmid pHLA-cadA using an In-fusion HD Cloning kit (TAKARABIO, Inc., Shiga, Japan). The resultant plasmid was named pHLA-cadA-blc-Tfu.

### Transformation of *E. coli*

Transformation of *E. coli* was carried out by electroporation with a 1350 kV, 600 Ω, 10 μF electric pulse in a 0.1 cm cuvette using a Gene Pulser (Bio-Rad Laboratories, Hercules, CA, USA). *E. coli* JCM20137 harboring pHLA-cadA, pHLA-blc-Tfu or pHLA-cadA-blc-Tfu was designated Jm-cadA, Jm-blc-Tfu and Jm-cadA-blc-Tfu, respectively.

### Western blotting

*E. coli* cells cultured at 37°C for 24 h were analyzed by sodium dodecyl sulfate-polyacrylamide gel electrophoresis (SDS-PAGE). Proteins were analyzed by SDS-PAGE using an SDS-polyacrylamide gel (10%; w/v). Dual color prestained Precision Plus protein standards (Bio-Rad Laboratories, Richmond, CA, USA) were used as molecular weight markers. The proteins were electroblotted onto a polyvinylidene difuluoride membrane (Millipore, Boston, MA, USA) and allowed to react with primary mouse anti-FLAG (Sigma) and secondary goat antimouse immunoglobulin G alkaline-phosphatase-conjugated antibodies (Promega Co., Madison, WI, USA). The membrane was then stained with nitroblue tetrazolium (NBT; Promega) and 5-bromo-4-chloro-3-indolylphosphate (BCIP; Promega) according to the manufacturer’s protocol.

### Measurement of BGL activity

Recombinant strains were cultured in 5 ml of LB medium containing 100 mg/l ampicillin. After incubation at 37°C for 24 h, the culture (2.5%; v/v) was used to inoculate 5 ml of R/2 medium containing 40 g/l cellobiose and 100 mg/l ampicillin, then the cells were grown at 37°C for 48 h. The BGL activity on the cell surface was quantitatively evaluated using p-nitrophenyl-β-D-glucopyranoside (pNPG; Nacalai Tesque, Inc., Kyoto, Japan) as a substrate. One unit of β-glucosidase activity was defined as the amount of enzyme producing 1 μmol/min p-nitrophenol at 37°C and pH 5.0.

### Fermentation experiments

Cadaverine fermentation by recombinant *E. coli* was performed in test tubes with a 5 ml working volume. R/2 medium containing 40 g/l glucose or 40 g/l cellobiose as the sole carbon source was used. *E. coli* cells grown on LB medium containing 100 mg/l ampicillin at 37°C for 24 h at 180 rpm were used to inoculate 5 ml of R/2 containing 40 g/l glucose or cellobiose at 37°C, 180 rpm. The initial OD600 was adjusted to 0.1. The initial pH of the medium was 6.8; the pH was not controlled during fermentation.

### Analytical methods

Throughout the 48 h of cultivation, BGL activities were measured as described above. Cell growth was monitored by measuring the OD600 with an UVmini-1240 UV–Vis spectrophotometer (Shimadzu, Kyoto, Japan). Cellobiose and glucose in culture supertnatants were analyzed using a Prominence HPLC System (Shimadzu) equipped with a SPR-Pb column (0.5 μm, 250 × 4.0 mm I.D.; Shimadzu). Water was used as the mobile phase at a flow rate of 0.6 ml/min, and the column was maintained at 80°C. The peak elution profile was monitored using a refractive index detector. The cadaverine concentration in the supernatant was analyzed using a TSKgel Polyaminepak (7 μm, 4.6 × 50 mm I.D.; TOSOH CORPORATION, Yamaguchi, Japan) using a Prominence Amino Acid Analyzer System (Shimadzu) after derivation with orthophthalaldehyde, according to manufacturer’s procedure. The mobile phase was a step gradient of a sodium citrate solution, pH 5.28, at a flow rate of 0.4 ml/min, and the column was maintained at 50°C, also according to manufacturer’s procedure.

## Results

### Expression of CadA in *E. coli*

**Figure 1:**
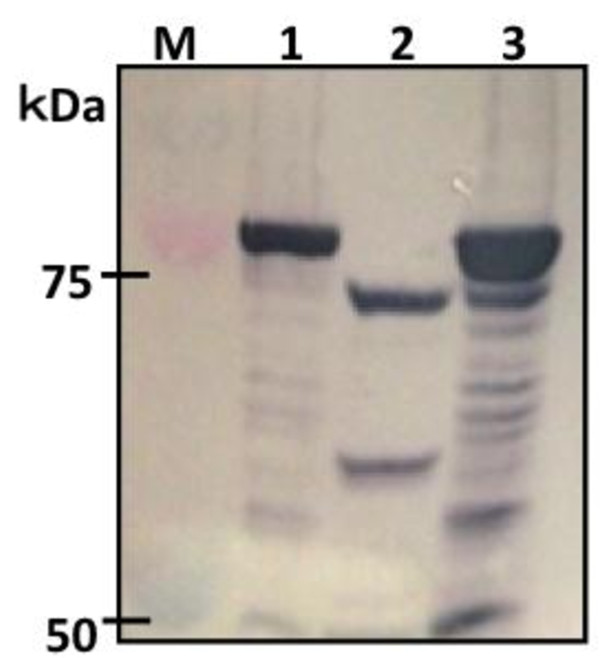
**Western blot analysis of CadA expression.** Lanes M: Marker protein with size indicated; lanes 1, 2 and 3: intracellular fractions of Jm-cadA, Jm-blc-Tfu and Jm-cadA-blc-Tfu, respectively.

Strains evaluated in this study are summarized in Table 1. Lysine decarboxylase (CadA) expression in *E. coli* is one of the important factors for cadaverine production. To confirm the expression of CadA in *E. coli*, intracellular fractions of *E. coli* cells were analyzed by Western blot analysis after 24 h of cultivation using glucose as a carbon source. Intracellular extracts of Jm-cadA and Jm-cadA-blc-Tfu showed a clear band corresponding to CadA-FLAG (82 kDa) (Figure 1, lanes 1 and 3). In contrast, no band was observed from extracts of Jm-blc-Tfu (Figure 1, lane 2). These results indicate the successful intracellular expression of CadA in Jm-cadA and Jm-cadA-blc-Tfu.

### BGL expression using *E. coli* as a host

BGL expression is needed for the efficient utilization of cellobiose. BGL activity on the cell surface of the BGL-expressing strains was quantitatively evaluated using pNPG as a substrate. As shown in Table 2, both of Jm-blc-Tfu and Jm-cadA-blc-Tfu showed higher BGL activity when grown on cellobiose (0.70 U/OD600/l of Jm-blc-Tfu and 0.74 U/OD600/l of Jm-cadA-blc-Tfu) compared to those grown on glucose (0.18 U/OD600/l of Jm-blc-Tfu and 0.13 U/OD600/l of Jm-cadA-blc-Tfu). No activity was detected on Jm-cadA cells. These results indicate that BGL was successfully expressed on the cell surface of Jm-blc-Tfu and Jm-cadA-blc-Tfu cells and retained its enzymatic function through the Blc anchor protein.

**Table 2:** BGL activities using Jm-cadA, Jm-blc-Tfu and Jm-cadA-blc-Tfu grown on glucose (40 g/l) or cellobiose (40 g/l) as a carbon source

**Strains**	**BGL activity grown on glucose (U/OD600/l)**	**BGL activity grown on cellobiose (U/OD600/l)**
Jm-cadA	Not detected	Not growth
Jm-blc-Tfu	0.18 ± 0.12	0.70 ± 0.03
Jm-cadA-blc-Tfu	0.13 ± 0.08	0.74 ± 0.07

### Cadaverine fermentation from glucose

**Figure 2:**
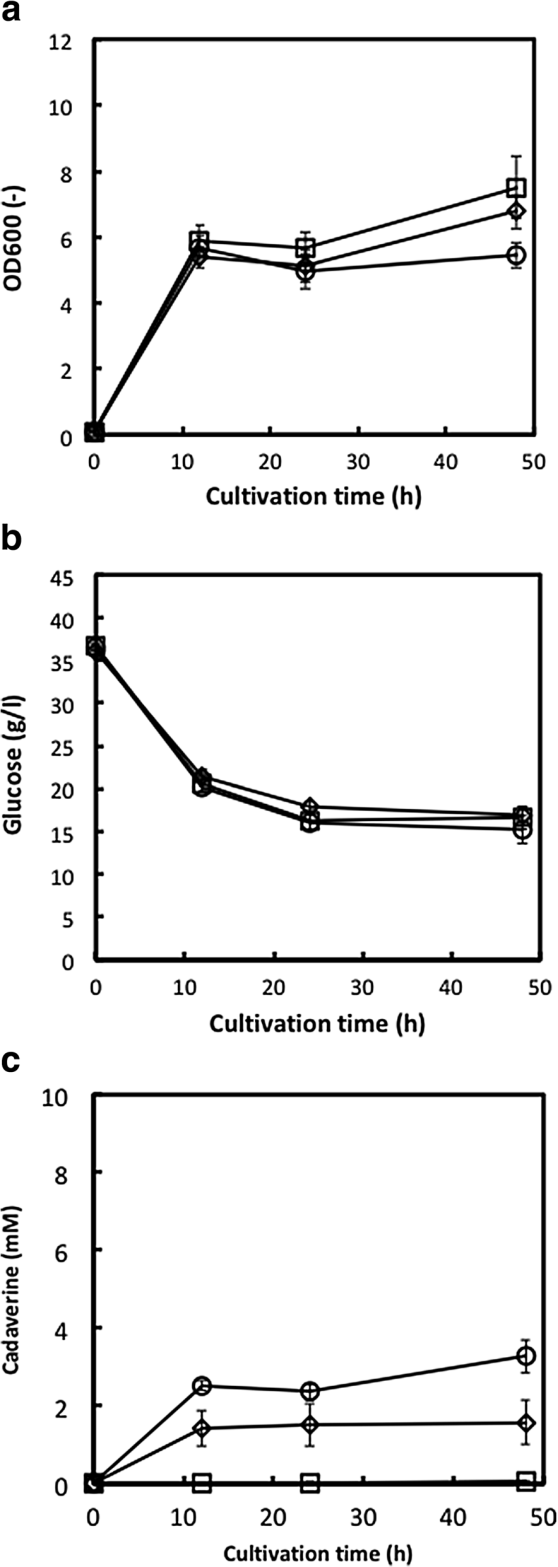
**Cadaveine fermentation experiments with glucose as the sole carbon source using Jm-cadA (diamonds), Jm-blc-Tfu (squares) and Jm-cadA-blc-Tfu (circles). (a)**, Cell growth measured by OD600, **(b)**, glucose concentration and **(c)**, cadaverine concentration. Data points represent means and standard deviation of three independent experiments.

**Figure 3:**
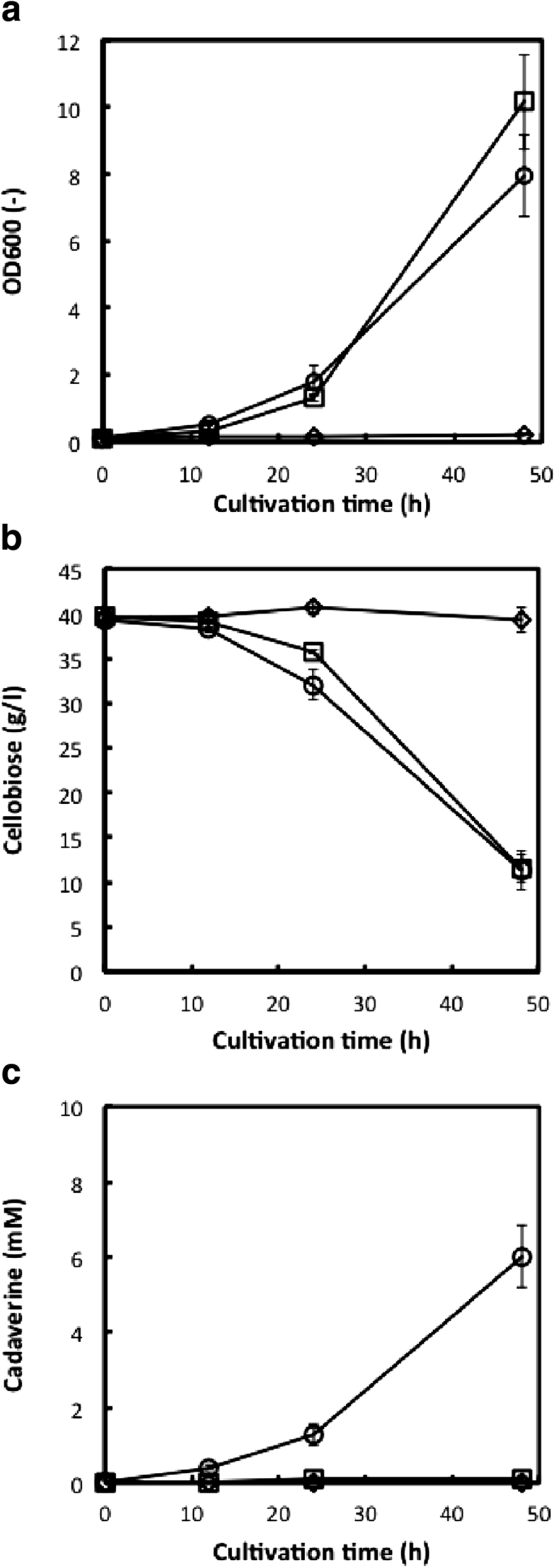
**Cadaveine fermentation experiments with cellobiose as the sole carbon source using Jm-cadA (diamonds), Jm-blc-Tfu (squares) and Jm-cadA-blc-Tfu (circles). (a)**, Cell growth measured by OD600, **(b)**, cellobiose concentration and **(c)**, cadaverine concentration. Data points represent means and standard deviation of three independent experiments.

We then evaluated cadaverine production by Jm-cadA, Jm-blc-Tfu and Jm-cadA-blc-Tfu grown in R/2 medium using glucose as the sole carbon source. Figure 2 shows the cell growth, glucose consumption and cadaverine concentration. As shown in Figure 3a, all strains showed similar growth, with OD600 values after 12 h cultivation of 5.4 (Jm-cadA), 5.9 (Jm-blc-Tfu) and 5.7 (Jm-CadA-blc-Tfu). The amount of consumed glucose after 24 h was 18.3 g/l (Jm-cadA), 20.3 g/l (Jm-blc-Tfu) and 20.3 g/l (Jm-CadA-blc-Tfu) (Figure 2b). As shown in Figure 2c, cadaverine production by the CadA-expressing strains was higher than by the control strain. The cadaverine concentration by Jm-cadA-blc-Tfu was 3.3 mM at 48 h, 1.5 mM by Jm-cadA and 0.1 mM by Jm-blc-Tfu. These results show that CadA-expression in *E. coli* resulted in higher cadaverine production.

### Cadaverine fermentation from cellobiose

We then evaluated cadaverine production using cellobiose as the sole carbon source. Figure 3 shows the cell growth, cellobiose consumption and cadaverine concentration using cellobiose as the sole carbon source in R/2 medium. The BGL-expressing strains, Jm-blc-Tfu and Jm-cadA-blc-Tfu, grew on cellobiose, providing OD600 values after 48 h cultivation of 10.1 (Jm-blc-Tfu) and 8.0 (Jm-cadA-blc-Tfu). Strain Jm-cadA, which does not express BGL, did not grow on cellobiose.

As shown in Figure 3b, BGL-expressing strains started consuming cellobiose after 24 h cultivation, with 28.2 g/l (Jm-blc-Tfu) and 28.0 g/l (Jm-cadA-blc-Tfu) cellobiose consumed after 48 h. Glucose was not detected in the culture medium during cultivation (data not shown).

The cadaverine concentration produced from cellobiose is shown in Figure 3c. No cadaverine was detected in the culture medium of Jm-cadA since it did not grow on cellobiose. In the case of Jm-blc-Tfu, only 0.1 mM of cadaverine was detected after 48 h, even though the strain grew on cellobiose (Figure 3b). In contrast, strain Jm-cadA-blc-Tfu produced 6.1 mM cadaverine after 48 h. This amount of cadaverine production is higher than that observed after 48 h of glucose fermentation by Jm-cadA-blc-Tfu.

## Discussion

The aim of this study was the direct production of cadaverine from cellobiose using *E. coli* expressing BGL and CadA. We previously reported the direct utilization of cellobiose using *E. coli* displaying BGL (Tanaka et al. 2011). Here, we engineered *E. coli* to produce cadaverine efficiency from cellobiose. This is the first report of cadaverine production using cellobiose as the sole carbon source by engineered *E. coli*.

Most wild-type *Escherichia coli* cells are unable to use cellobiose due to the cryptic nature of the cellobiose-utilizing operons such as bgl and cel (Parker and Hall 1990). There are two pathways for cellobiose utilization in *E. coli*. One is the direct import of cellobiose through the PTS (PEP-dependent phosphotransferase system). Indeed, it was reported that a cellobiose-positive phenotype could be easily obtained by prolonged cultivation using cellobiose as the sole carbon source (Hall and Betts 1987; Keyhani and Roseman 1997; Parker and Hall 1990). Efforts to engineer *E. coli* to use cellobiose are of interest as commercial cellulase cocktails lack sufficient beta-glucosidase activity, which makes supplementation with beta-glucosidase necessary (Xin et al. 1993). Alternatively, expressing the cel operon from *Klebsiella oxytoca* in *E. coli* allows cellobiose to be transported through a phosphotransferase system to yield intracellular cellobiose-phosphate, which is subsequently cleaved by another operon-encoded enzyme, phospho-beta-glucosidase, to yield glucose-6-phosphate and glucose (Moniruzzaman et al. 1997).

The other approach is the heterologous expression of BGL and the conversion of cellobiose to glucose. Although the degradation of cellobiose is the rate-limiting step, and Tfu0937 described in this study currently exhibits insufficient BGL activity, the expression of a suitable BGL and optimization of fermentation conditions will improve productivity. The expression of beta-glucosidase and its display on the cell surface has been attempted and resulted in some reduction in the need for BGL (Ha et al. 2011).

In this study, we constructed BGL- and CadA-expressing *E. coli*. The BGL activity of Jm-blc-Tfu and Jm-CadA-blc-Tfu grown on cellobiose was higher than those grown on glucose (Table 2). Although all *E.coli* strains can be grown on glucose, the suitable E.coli strain (i.e. having high BGL activity) was selected during the cultivation using cellobiose as a carbon source. No glucose was detected in the culture medium during fermentation, suggesting that the rate-limiting step was cellobiose hydrolysis; if this is the case, then improving BGL activity on the cell surface would lead to cadaverine production. That the growth rate with glucose as the carbon source was higher than when cellobiose was the carbon source also suggests that increasing the glucose concentration produced by BGL from cellobiose would improve cadaverine production.

CadA expression in *E. coli* increased the amount of cadaverine produced, from 0.1 mM up to 3.3 mM, using glucose as the carbon source (Figure 2). The utility of CadA overexpression for cadaverine production from glucose has been previously demonstrated; for example, 12.8 mM in *E. coli* (Qian et al. 2011) and 50 mM in *C. glutamicum* (Tateno et al. 2009). Metabolic engineering of the cadaverine production pathway is also a promising approach for increasing cadaverine production. Na et al. (2013) demonstrated cadaverine production using metabolic pathway engineered *E. coli* and obtained up to 21.1 mM cadaverine. Pathway engineering of *C. glutamicum* also improved cadaverine productivity (Buschke et al. 2013a, b; Kind et al. 2011). In this study, we demonstrated direct cadaverine production from cellobiose using a BGL-displaying and cadA-expressing *E. coli* strain. Interestingly, when cellobiose was used as the sole carbon source, the OD600 values were higher than when glucose was used as the sole carbon source (Figures 2a and 3a). Additionally, the amount of cadaverine produced from cellobiose was higher than from glucose (Figures 2c and 3c), although the growth rate, sugar consumption rate and cadaverine production rate from cellobiose were lower than when the cells were grown on glucose. It is possible that the sugar concentration around the cells may affect metabolism; if so, than providing controlled amounts of glucose to the culture may help improve cadaverine production. However, sugar consumption and cadaverine production were prevented after 24 h fermentation (Figures 2 and 3), which was possibly caused by low pH (about 4.0 after 48 h cultivation). Controlling pH during fermentation may be improved cadaverine productivity, as well as metabolic engineering.

In conclusion, we have demonstrated the direct production of cadaverine from cellobiose using BGL-displaying and CadA-expressing *E. coli*. Active BGL Tfu0937 was displayed on the cell surface and direct cadaverine fermentation from cellobiose was demonstrated. Our results indicate that cellobiose is a more suitable substrate for *E. coli* expressing BGL than glucose in some cases. For the direct utilization of cellulose, other kinds of cellulases, such as EG and CBH, are required. To address this, we are currently constructing *E. coli* coexpressing cellulases for the direct and efficient production of cadaverine from cellulose.

## Competing interests

The authors declare that they have no competing interests.
